# The role of immunotherapy in non-clear cell renal cell carcinoma

**DOI:** 10.3389/fonc.2023.941835

**Published:** 2023-02-03

**Authors:** Carla Climent, Sandra Soriano, Teresa Bonfill, Natalia Lopez, Marta Rodriguez, Marina Sierra, Pablo Andreu, Monica Fragio, Mireia Busquets, Alicia Carrasco, Ona Cano, Miguel-Angel Seguí, Enrique Gallardo

**Affiliations:** Department of Medical Oncology, Consorcio Hospital Universitario Parc Tauli, Sabadell, Spain

**Keywords:** renal cancer, non-clear cell renal cell carcinoma, immunotherapy, PD-L1, sarcomatoid differentiation

## Abstract

The category of non-clear cell renal cell carcinoma (nccRCC) includes several clinically, histologically, and molecularly diverse entities. Traditionally, they comprise type 1 and type 2 papillary, chromophobe, unclassified, and other histologies (medullary, collecting duct carcinoma, and translocation-associated). Molecular knowledge has allowed the identification of some other specific subtypes, such as fumarate hydratase–deficient renal cell carcinoma (RCC) or succinate dehydrogenase–associated RCC. In addition, it has recognized some alterations with a possible predictive role, e.g., MET proto-oncogene receptor tyrosine kinase (MET) alterations in papillary tumors. Standard therapies for the management of advanced clear cell RCC (ccRCC), i.e., vascular endothelial growth factor receptor (VEGFR) pathway inhibitors and mammalian target of rapamycin inhibitors, have shown poorer results in nccRCC patients. Therefore, there is a need to improve the efficacy of the treatment for advanced nccRCC. Immunotherapy, especially immune checkpoint inhibitors (ICIs) targeting programmed death 1/programmed death ligand 1 and cytotoxic T-lymphocyte associated protein 4 (CTLA-4), has demonstrated a significant survival benefit in several malignant neoplasias, including ccRCC, with a proportion of patients achieving long survival. The combinations of ICI or ICI + VEGFR tyrosine kinase inhibitors (TKIs) are the standard of care in advanced ccRCC. Unfortunately, major pivotal trials did not include specific nccRCC populations. In recent years, several studies have retrospectively or prospectively evaluated ICIs alone or in combination with another ICI or with TKIs in nccRCC patients. In this article, we review data from available trials in order to elucidate clinical and molecular profiles that could benefit from immunotherapy approaches.

## Introduction

Kidney cancer is responsible for 2%–3% of all malignant tumors in adults. The relative survival rate at 5 years differs depending on whether the kidney cancer is localized or advanced (93% and 12%, respectively). Among diagnosed renal tumors, clear cell renal cell carcinoma (ccRCC) is the most frequent type, representing 80%; the remaining 20% are non-clear cell renal cell carcinoma (nccRCC), a group of rare and histopathologically heterogeneous tumors (1). In most kidney cancer studies, nccRCC tumors are not included or are very poorly represented and, for this reason, little is known about the best management for these subtypes.

A retrospective study of metastatic renal cell carcinoma (RCC) patients (42,35 ccRCC and 337 nccRCC) who received targeted therapies found that, first, median overall survival (OS) was shorter for those patients with nccRCC than with ccRCC [15.7 vs. 20.2 months; hazard ratio (HR), 1.41]. Second, this study highlighted that patients with nccRCC who had received VEGF-targeted therapy had shorter median progression-free survival (PFS) than patients with ccRCC (6.1 vs. 8.5 months; HR, 1.49; P < 0.001), but median PFS was similar when treated with mammalian target of rapamycin (mTOR) inhibitors (4.3 vs. 4.4 months; HR, 0.92; P = 0.63) (2).

There are several subtypes of nccRCC: papillary RCC (PRCC), representing 10%–15% and being a unique entity since the last WHO classification published in 2022, where it is recommended not to divide PRCC into type 1 and 2 as was done in the previous classification of 2016: chromophobe RCC (chRCC), representing 5% and has a low risk of metastasis; collecting duct carcinoma (CDC), representing 1% and has a poor prognosis; medullary carcinoma, representing 1%, is aggressive, and has a poor prognosis; and Xp11.2 translocation, representing 1%, is aggressive, and has a poor prognosis) (3–5).

New, less frequent nccRCC subtypes were described in the 2016 WHO classification, for instance, succinate dehydrogenase B-deficient, hereditary leiomyomatosis, and syndrome-associated RCC with fumarate hydratase deficiency (6). Sarcomatoid RCC has not been defined as a distinct entity as all RCCs can exhibit sarcomatoid differentiation.

Not only were patients histopathologically different but also molecular profile differences were recently described for nccRCC. These differences could allow test trials with targeted systemic therapies according to the molecular mutation detected. For PRCC, the most frequent mutation is in *MET* (especially in type 1), PRCC type 2 being associated with *CDKN2A* silencing, *SETD2* mutations, *TFE3* fusions, and increased expression of the mammalian target of rapamycin Nuclear factor erythroid 2-related factor 2 (NRF2)-antioxidant response element pathway. Regarding chRCC, the most frequent mutations are in *TP53*, *PTEN*, and genes located at the short arm of chromosome 7. As for CDC, its genomic profile shows mutations in *NF2*, *SETD2*, *SMARCB1*, *CDKN2A*, and *SLC7A11* (7–9).

As previously mentioned, treatment options for nccRCC are limited as there are fewer specific studies available. However, some studies compared TKI and mTOR therapies for nccRCC. On the one hand, the ASPEN, ESPN, and RECORD-3 trials compared sunitinib and everolimus; of these, only the RECORD-3 trial included patients with ccRCC, which was the most frequent subtype in both treatment groups (85% and 86% of total patients, respectively). In the three studies, sunitinib showed better benefits than everolimus in PPRC; however, in chRCC, everolimus showed to be more beneficial (10–12). On the other hand, the CESAR study group published a trial comparing temsirolimus and sunitinib (13). Of the 22 patients included, 73% were PRCC; no chRCC was included in the sunitinib arm. Furthermore, for PRCC, the benefit was greater with sunitinib.

In the specific case of PRCC, TKIs with action on MET have been evaluated. The PAPMET trial was the first randomized study for PRCC where four different TKIs with an inhibitory effect on MET were tested: cabozantinib, crizotinib, savolitinib, and sunitinib. The crizotinib and savolitinib arms closed prematurely. This trial demonstrated longer PFS for cabozantinib treatment than for sunitinib (9 vs. 5.6 months, respectively; HR 0.60, p = 0.019) (14).

In addition to the molecular profile, increasing evidence has shown that the tumor microenvironment (TME) also plays a crucial role in targeted drug efficacy. Gradually, it has been recognized that tumor-infiltrating cells can affect the response to treatment or aggravate drug resistance in the TME (15). In addition, and thanks to recent advances in understanding the biology of neoplastic cells, it is now known that these cells can escape immunological responses by, for example, expressing ligands to block immunomodulatory cell receptors. Some new treatments prevent cancer cells from blocking the immunomodulatory cell receptors implicated in the immune response. Here, the activity of immune cells, such as effector T cells, B lymphocytes, macrophages, and natural killer cells, increases. Some well-described immune checkpoints are programmed death 1 (PD-1) and CTLA-4 (cell surface proteins expressed in immune cells) and PD-L1 (cell surface protein expressed in some tumoral cells to varying degrees). The expression of PD-L1 in nccRCC varies according to the subtype. In the last years, the role of PD-1 and its ligand [programmed death ligand 1 (PD-L1)] has been studied in nccRCC patients; although the prognostic value of PD-L1 positivity in nccRCC remains unclear, it seems that PD-L1 expression in nccRCC is related to tumor aggressiveness (16–19).

During the last years, the diagnosis, management, and treatment of the ccRCC subtype have seen improvements resulting from various randomized and prospective phase III clinical trials including immunotherapy, which acts on immune checkpoints such as PD-1, CTLA-4, and PD-L1 (Javelin 101, CheckMate 9ER, Keynote 426, CheckMate 214, and CLEAR) (20–26).

nccRCC treatments are based on those established for ccRCC since there are few specific studies for this population and the ccRCC trials did not include nccRCC subtypes (27). This review aims to resolve or clarify the role of checkpoint inhibitors in patients with nccRCC and discern those patients who could better benefit from this therapy.

## Immunotherapy treatment

### Immune checkpoint blockade monotherapy

#### Nivolumab

Nivolumab is a humanized monoclonal anti-PD1 antibody approved for various metastatic solid tumors. For metastatic RCC (mRCC), the approval was based on data from CheckMate-025. In this prospective phase III trial, nivolumab showed higher OS compared with everolimus (25 vs. 19.6 months, HR 0.73, P = 0.002), and a better overall response rate (ORR) (25% vs. 5% P < 0.001) for patients with ccRCC refractory prior to antiangiogenic therapy (28, 29). The trial did not include patients with nccRCC; however, the treatment was approved for these subtypes.

The evidence of nivolumab’s benefits in ccRCC motivated clinical trials evaluating it in nccRCC. In 2018, the first retrospective study including 41 nccRCC patients receiving at least one dose of nivolumab was published. Histology included 16 PRCC (39%), 14 unclassified (34%), 5 chRCC (12%), and 4 CDC (10%). Of these, 12% presented sarcomatoid components and 62% received one or more lines of treatment before the study. The median follow-up time was 8.5 months, the ORR was 20%, and all were partial responses (PRs). Responses were observed in 36% unclassified, 25% CDC, 14% PRCC, and 0% chRCC. Median PFS was 3.5 months, and median OS was not reached at the time of analysis (30) (**Table 1**). The ORR observed in this trial was equivalent to that of the CheckMate-025 trial in ccRCC (29, 30). Despite the differences in the biology and underlying molecular mechanisms between ccRCC and nccRCC, the results of this trial show that immune checkpoint inhibitors (ICIs) might have a potential benefit in both groups.

**Table 1:** Monotherapy inmune-checkpoint blockade.

Treatment	Type of trial	Population	Sarcomatoid	PD-L1 ≥1	ORR	PFS (months)	OS (months)	Adverse events ≥G3
Nivolumab (30)	Retrospective	N= 41 39% P 34% UC 12% Ch 10% CDC	12%	NA	20% overall? 14% P 36% UC 0% Ch 25% CDC	3,5	NA	24.4% Fatigue Fever Rash / Skin Toxicity
Nivolumab Checkmate-374 (31)	Prospective	N= 44 54,5% P 18,2% UC 15,9% Ch 5% Translocation 2% CDC 2% Medular	9%	34,2%	13,6% overall	2,2	16,3	No grade 3-5 treatment-rel ated IMAEs occurred
Nivolumab (32)	Prospective	N= 50 51% P 18% Ch 8% CDC 8% UC	< 15%	Not reported	10% overall 16% P 0% Ch CDC 25% others 12,5%	NR (Dec 2023)	NR (Dec 2023)	Not reported (Dec 2023)
Pembrolizumab (33)	Prospective	N= 165 71,5% P 15,8% UC 12,7% Ch	23%	61%	26.7% overall 28% P 9,5% Ch 30,8% UC	4,2	28,9	17% Colitis Fatigue Cough Hepatitis

NA, not applicable; NR, not reached; P, papillary; UC, Unclassified; Ch, Chromophobe; CDC, Collecting ducts; OS, overall survival; PFS, progression-free survival; ORR, overall response rate. Trials for nccRCC with nivolumab and pembrolizumab as monotherapy showed heterogeneous ORR results, with patients with unclassified histology having the highest response rate, followed by CDC, PRCC, and chRCC.

In light of such findings, in 2020, Chahoud et al. performed a meta-analysis across three studies with 124 nccRCC patients treated with nivolumab. The ORR [18.6%, including 8.8% with complete remission (CR)] was homogeneous and consistent across studies and similar to the previous retrospective trial (34) (**Table 1**).

After the benefits seen for nivolumab in retrospective nccRCC studies, the first prospective phase IIIb/IV trial, CheckMate-374 (NCT02596035), was carried out to evaluate flat-dose nivolumab monotherapy 240 mg every 2 weeks (Q2W) in patients with ccRCC, nccRCC, or brain metastases. The study included 44 patients with nccRCC: 24 PRCC (54.5%), 8 unclassified (18.2%), 7 chRCC (15.9%), 2 translocation-associated (5%), 1 CDC (2%), 1 medullary (2%), and 1 unreported subtype. Sarcomatoid differentiation was present in 9.1% of patients, and 65.9% received no prior systemic treatment. PD-L1 expression was evaluated using PD-L1 tissue immunohistochemistry (IHC), and 34.2% of patients were PD-L1≥ 1%.

Median follow-up was 11 months; the ORR was 13.6%. The most frequent response was disease progression (40.9%), followed by stable disease (36.4%) and PR (11.4%). One patient with chromophobe histology and no prior systemic therapy achieved a CR (2.3%). Median PFS was 2.2 months. PD-L1 was neither prognostic nor predictive of efficacy, and significant ORR results were observed regardless of baseline PD-L1 expression. Nonetheless, patients with positive PD-L1 presented better OS than those with negative PD-L1 (16.3 vs. 11.8 months) (31) (**Table 1**).

No grade 3–5 treatment-related adverse effects were observed. The safety profile observed in the CheckMate-025 study with nivolumab at 3 mg/kg Q2W remained consistent with that observed in flat-dose nivolumab at 240 mg Q2W (29, 31).

A second, ongoing prospective trial with nivolumab in nccRCC patients, the secured access (AcSe) study (NCT03012581), evaluated the safety and efficacy of nivolumab in patients with specific rare cancers. The nccRC cohort included 50 patients [20 PRCC type 2 (41%), 9 chRCC (18%), 5 PRCC type I (10%), 4 unclassified (8%) 4 CDC (8%), and 8 patients with other subtypes]; 16% of patients were treatment-naïve, and 54% had only received one line of treatment.

The ORR was 10% (11% for PRCC type 1, 5% type 2, 25% CDC, 0% chRCC, and 12.5% others) (32) (**Table 1**). Further results regarding median PFS and median OS are pending (December 2023).

#### Pembrolizumab

Keynote-427 (NCT02853344), published in 2021, was a prospective phase II single-arm study using the anti-PD-1 inhibitor, pembrolizumab, in patients with mRCC, composed of two cohorts for ccRCC and nccRCC.

Cohort B included 165 patients with nccRCC [118 PRCC (71.5%), 26 unclassified (15.8%) and 21 chRCC (12.7%)], 23% of whom presented sarcomatoid differentiation. PD-L1 was determined by a combined positive score and was positive in 61.8% of cases. All patients were treatment-naïve. The ORR was 26.7% (30.8% unclassified, 28% PRCC and 9.5% chRCC), and the unclassified histology presented the greatest CR rate compared with PRCC and chRCC (11.5%, 5.9%, and 4.8%, respectively). Median PFS and OS were 5.5 and 31.5 months for PRCC, 3.9 and 23.5 months for chRCC, and 2.8 and 17.6 months for unclassified.

In this study, patients expressing a PD-L1 ≥ 1 had a higher ORR compared with patients with PD-L1 < 1 (35.3% vs. 12.1%), with higher CR (7.8% vs. 5.2%) and PR rates (27.5% vs. 6.9%) (33) (**Table 1**).

Considering prospective trials, the comparison of data is difficult due to differences between studies. Pembrolizumab achieved higher PFS and OS than nivolumab in Checkmate-374 (4.2 vs. 2.2 months and 28.9 vs. 16 months, respectively) and a higher ORR than nivolumab (26.7% with pembrolizumab vs. 13.6% or 10% with nivolumab), but the studies presented differences in the design and the population included. The nivolumab study included previously treated patients, and the pembrolizumab study included all treatment-naïve patients. Moreover, the subtypes included in each study and their representation percentage were different. Because of that, the subtype with the better response to immunotherapy is different in each trial because the populations studied were heterogeneous. The papillary subtype was highly present in both studies, and pembrolizumab showed a more significant response rate than nivolumab (28.8% with pembrolizumab vs. 12.5% or 11% with nivolumab) in that subtype (31–33).

### Combination therapy

#### Immune checkpoint inhibitor + immune checkpoint inhibitor

##### Ipilimumab + nivolumab

Ipilimumab + nivolumab, the combination of two monoclonal antibodies targeting anti-CTLA4 and anti-PD-1, respectively, was recently approved to treat International Metastatic RCC Database Consortium (IMDC) intermediate- and poor-risk metastatic ccRCC after CheckMate-214, where the combination ICI + ICI showed benefits in PFS (HR 0.76) and OS (HR 0.66) in comparison with sunitinib (24). Updated results showed a 42-month OS rate of 50% with ipilimumab + nivolumab vs. 36% with TKIs and a better ORR for the combination (42% vs. 26%), including CR (10% vs. 1%) (25). Unfortunately, this trial only included patients with a clear cell component, excluding nccRCC patients.

Nonetheless, there have been previous retrospective studies in nccRCC patients. In 2019, a retrospective study was published, where the clinical activity of ipilimumab + nivolumab was evaluated in 18 patients with metastatic nccRCC. The population included six patients with PRCC (33%), five chRCC (28%), three unclassified (18%), two adenocarcinoma (12%), one translocation (5%), and one with medullary histology (5%). The most frequent IMDC risk group was intermediate (66%) or poor (22%), and 72% of patients were treatment-naïve. The ORR was 33.3%: three PRCC (50%), one adenocarcinoma (50%), one unclassified (33%), and one chRCC (20%). There were 50% of patients who had progressive disease as the best response, and 17% presented stable disease. Median PFS was 7.1 months (35) (**Table 2**).

**Table 2:** Combination therapy.

Treatment	Type of Trial	Population	Sarcomatoid	PD-L1 ≥ 1	ORR	PFS(months)	OS(months)	Adverse events≥G3
Ipilimumab+Nivolumab (35)	Retrospective	N= 18 33% P 28% Ch 17% UC 12% ADK 5% Translocation 5% Medular	11.11%	NA	33.3% overall 50% P 20% Ch 33.3% UC 0% Translocation 0% Medular	7.1	At 12months:64.2%	38% Colitis Hepatitis Hypophysitis Myopathy Encephalitis
Ipilimumab+Nivolumab (36)	Retrospective	N= 33 30% P 30% UC 10% Translocation 10%Ch 10% SC	20%	NA	30% overall	3.5	19.6	30% Hepatic failure Pneumonitis Hypopituitarism Adrenalin sufficiency
Ipilimumab+Nivolumab Checkmate-920 (37)	Prospective Single arm	N= 52 42.3% UC 34.6% P 13.5% Ch 3.8% Translocation 3.8% CDC 1.9% Medular	28%	38.5%	19.6% overall PDL1≥1: 30.8% PD-L1<1: 14.3% Sarcomatoid differentiation: 35.7%	3.7	21.2	36.5%ColitisRashNephritis
Atezolizumab+Bevacizumab (38)	Prospective Single arm	N= 60 k/>30% ccRCCSd 70% nccRCC: 35% P 29% Ch 26% UC 15% CDC 15% Translocation 3% Medular	30%	42%	34% overall 53% ccCRRSd 26% nccRCC 40% CDC 33% UC 25% P 10% Ch PDL1≥1: 64% PD-L1<1:20%	8.4	NR	56.6% Diarrhea Proteinuria Hypertension Hypertension Mucositis
Atezolizumab+Cabozantinib COSMIC-021 (39)	Prospective Single arm	N=102 68,6% ccRCC 31,4 nccRCC 47% P 28% Ch 22% Others	26% in the 40-mg cabozantinib ccRCC cohort 6% in the 60-mgcabozantinib ccRCC cohort 13% in nccRCC cohort	26% in the 40-mg cabozantini b ccRCC cohort 22% in the 60-mg cabozantini b ccRCC cohort 13% in nccRCC cohort	53% in the 40-mg cabozantinib ccRCC cohort 58% in the 60-mg cabozantinibccRCC cohort ORR was 31% in nccRCC cohort 47% P 11% Ch Other 25% ORR independent of the PD-L1 status	19.5 in the 40-mgcabozantini b ccRCC cohort 15.1 months in the 60-mg cabozantini b ccRCC cohort 9.5 months in nccRCCcohort	No reported(Dec2022)	71% patient in the 40-mg ccRCC cohort 67% in the 60-mg ccRCCcohort 38% in the nccRCC cohort Hypophosphatem ia Hypertension ALT increased PPELipase increased
Nivolumab+Cabozantinib (40)	Prospective Single arm	N= 47 Cohort 1: 80% P 15% UC 5% TranslocationCohort 2: 100% Ch (7 patients)	4.2%	NA	Cohort 1: 47,5% 47% P 50% UC 50% Translocation Cohort 2: 0%	Cohort 1:12.5 Cohort 2: NA	Cohort 1:28 Cohort 2: NA	32% Hypertension Diarrhea PPE
Durvalumab+Savolitinib CALYPSO (41)	Prospective Single arm	N= 41 100% P	Not reported (Dec 2022)	ORR overall Not reported (Dec 2022)7% in MET-driven group	29% overall 57% in MET-driven group	4.9 overal 10.5 in MET-driven group	12.9overall 27.4 in MET-driv en group	Not reported (December 2022)

NA, not applicable; NR, not reached; P, papillary; UC, Unclassified; Ch, Chromophobe; CDC, Collecting ducts; ccRCCSd, clear cell renal carcinoma with sarcomatoid differentiation; nccRCC, non clear cell renal carcinoma; ADK, Adenocarcinoma; SC, Spindle cell subtype; OS, overall survival; PFS, progression-free survival; PPE, palmar-plantar erythrodysesthesia syndrome; Dec, December

As for the trials with an ICI + ICI combination, the retrospective trials indicated that the ICI combination presented a better response rate than nivolumab alone (ORR 30-33.3% vs. 13.6%) (31, 35, 36). On the contrary, the prospective study Checkmate-920 showed less benefit than retrospective trials but without being a comparative study with the rest, since it targeted patients with characteristics that conferred a worse prognosis (low Karnofsky grading and brain disease) (37). Regarding the ICI + Target therapy combination, the population included in the different studies is small. In all of these trials, the most represented histology was PRCC, but the proportion of PRCC compared with other subtypes in the studies was different and made it difficult to draw any clear conclusions. For PRCC, combinations containing a MET-specific TKI (savolitinib) or a multi-TKI targeting MET (cabozantinib) had the best response rates (38–41). Regarding the other histologies, their representation was heterogeneous in the studies. In the combination studies, CDC patients are not represented in all the trials, and the trial with more CDC patients was atezolizumab + bevacizumab, with five patients, with an ORR of 40% (38). It is known that CDC is related tohigh lymphocyte infiltration into the tumor and higher PD-L1 expression compared with other subtypes. This should motivate a more direct study of the benefit of immunotherapy in these patients. In the case of unclassified subtypes, combination therapies, the trials providing direct data for unclassified subtypes were the nivolumab + cabozantinib (which included six patients with an ORR of 50%) and atezolizumab + bevacizumab studies (which included nine patients with an ORR of 33%) (38, 40). Consideringthat the responses obtained with other treatments, such as TKI or mTOR, range from 0% to 13%, immunotherapy does seem to have a clear benefit in this patient subtype. chRCC is represented in all combination therapy trials with a low ORR, ranging from 0% with nivolumab + cabozantinib to 11% with atezolizumab + cabozantinib. The fact that this subtype is associated with low PD-L1 expression could explain the poorer response to immune checkpoint inhibitor therapies. Thus, immunotherapy does notseem the best option and it would be necessary to carry out more targeted studies, despite the low incidence.

In 2022, Japanese scientists published a new retrospective study evaluating treatment with ipilimumab + nivolumab vs. treatment with TKIs or mTOR in 33 patients with nccRCC. The ipilimumab + nivolumab cohort included only 10 patients: three PRCC (30%), three unclassified (30%), two translocation–association (20%), one chRCC (10%), and one spindle cell subtype (10%). The ORR rate was 30% in the combination cohort (p=0.04); all were CR (two PRCC patients and one with the spindle subtype). In the TKI/mTOR cohort, the ORR was 4.3% with only PRs. Despite this significant benefit in response, the benefit in OS [19.6 with ICI + ICI vs 10.6 months with TKI/mTOR (p=0.23)] and PFS [3.5 months with ICI + ICI vs 4.7 months with TKI/mTOR (p=0.61)] was not significant (36) (**Table 2**).

The retrospective trials indicated that the ICI combination presented a better response rate than nivolumab alone (ORR 30%–33.3% vs. 13.6%). As for adverse effects, the ICI combination presented more (61% vs. 37%) than nivolumab alone (31, 35). Nonetheless, results were not evaluated for PD-L1 expression in either combination trial.

The data obtained in retrospective studies encouraged further prospective trials. Preliminary data from the phase IIIb/IV Checkmate-920 prospective trial (NCT02982954) have recently been published. This single-arm trial evaluated the role of ipilimumab + nivolumab in previously untreated patients who were often excluded from other studies, such as patients with nccRCC, brain metastases, or patients with poor performance status.

This study consisted of four cohorts. Cohort 1: ccRCC with Karnofsky performance status ≥ 70%. Cohort 2: nccRCC with Karnofsky performance status ≥ 70%. Cohort 3: nccRCC/ccRCC with asymptomatic brain metastases. Cohort 4: nccRCC/ccRCC with Karnofsky performance status 50%-60%. The primary endpoint was the incidence of any-causality grade ≥ 3 immune-mediated adverse events.

The study enrolled 52 nccRCC patients; histologies included 22 unclassified (42.3%), 18 PRCC (34.6%), 7 chRCC (13.5%), 2 translocation-associated (3.8%), 2 CDC (3.8%), and 1 renal medullary (1.9%). Sarcomatoid differentiation was present in 28% of patients, and 38.5% were PD-L1 positive. Median follow-up was 26 months.

The ORR was 19.6%, with two CRs (one PRCC and one unclassified patient) and seven PRs (four PRCC and three unclassified). Patients with positive PD-L1 had a better response than PD-L1-negative patients (30.8% vs. 14.3%). The group with sarcomatoid differentiation obtained a higher response rate than patients without (35.7% vs. 12.5%). Median PFS was 3.7 months, and median OS was 21.2 months. Regarding the primary endpoint, no grade 5 adverse reactions were described; the most frequent grade 3–4 reactions were colitis (7.7%), rash (5.8%), and nephritis (3.8%) (37) (**Table 2**).

Comparing cohorts 1 (ccRCC) and 2 (nccRCC), the groups were not correctly balanced. There were twice as many ccRCC patients as nccRCC (106 vs. 46, respectively). With these limitations, ipilimumab + nivolumab presents a lower response rate in patients with nccRCC than ccRCC. However, in both groups, patients with positive PD-L1 showed better responses than patients with negative PD-L1. More investigation is necessary to confirm that PD-L1 is a biomarker to predict response to combination ICI + ICI therapy.

#### Immunotherapy (IO) + tyrosine kinase inhibitor

The beneficial results obtained in clinical studies with immunotherapy treatment afforded the opportunity to combine such therapies with others using different mechanisms to enhance the immunomodulatory effects (42).

Some TKIs could potentially alter the TME by increasing T-lymphocyte infiltration into the tumors, thus increasing the sensitivity of the immune checkpoint blockade (43, 44). Several trials evaluated TKI/ICI combination therapies in ccRCC and showed improved ORR, PFS, and OS vs. standard of care (sunitinib). These studies were Checkmate-9ER (cabozantinib + nivolumab), Keynote-426 (axitinib + pembrolizumab), and CLEAR (lenvatinib + pembrolizumab) (21, 22, 26), all focusing on ccRCC and not including less frequent subtypes of renal cancer. For this reason, such treatments are not yet approved for nccRCC. During the last years, new prospective trials have been carried out to evaluate the efficacy of the TKI/ICI combination in less frequent subtypes.

##### Atezolizumab + bevacizumab

The IMmotion151 study compared atezolizumab + bevacizumab vs. sunitinib in ccRCC patients. The combined therapy showed benefit over TKIs for PFS (HR 0.74 p=0.02) but not OS (HR 0.93) (45, 46). Yet, as nccRCC appears to have a worse prognosis than ccRCC, combined therapies might provide better results in nccRCC than in ccRCC.

According to this hypothesis, in 2019, a phase II single-arm study (NCT02724878) with atezolizumab + bevacizumab was conducted in patients with nccRCC and ccRCC with >20% sarcomatoid differentiation.

The study included 60 patients (18 ccRCC with sarcomatoid differentiation and 42 nccRCC). The nccRCC cohort consisted of 12 PRCC (35%), 10 chRCC (29%), 9 unclassified (26%), 5 translocation-associated (15%), 5 CDC (15%), and 1 medullary subtype (3%); 52% of patients were treatment-naïve. PD-L1 was evaluated by double IHC using two antibodies against PD-L1 (anti-CD45 and anti-CD163); 42% had PD-L1 ≥ 1%. The ORR was 34% (53% in ccRCC and 26% in nccRCC), being higher in CDC (40%) and unclassified (33%) than in PRCC (25%) and chRCC (10%). Patients with PD-L1 ≥ 1% were associated with an improved ORR (64%) compared with those with PD-L1 < 1% (20%). Median PFS was 8.4 months, and median OS was not available at the time of the analysis (38) (**Table 2**).

In the IMmotion 151 trial for ccRCC, the ORR was similar to that of the nccRCC trial (37% and 34%, respectively) and, in both trials, patients with PD-L1 ≥ 1% had a higher response (38, 45).

##### Atezolizumab + cabozantinib

In nccRCC, the best results with a TKI in monotherapy were obtained with cabozantinib. The PAPMET trial, a phase II randomized study evaluating cabozantinib vs. sunitinib in PRCC, demonstrated improvements in PFS (HR 0.6 p=0.019) and the ORR (p=0.010) with cabozantinib (14). After several studies, cabozantinib was shown to promote an immune-sensitive tumor environment that could enhance the immune response by mitigating immunosuppression and promoting tumor cytotoxic T-cell infiltration (47–49). These studies motivated the evaluation of the combination of cabozantinib with immunotherapy in prospective trials for nccRCC.

COSMIC-021 (NCT03170960), a multicohort basket phase Ib single-arm trial, evaluated the ORR of cabozantinib + atezolizumab and included patients with 12 different types of solid tumors, including 70 patients with ccRCC and 32 with nccRCC [15 PRCC (47%), 9 chRCC (28%), and 7 other subtypes (22%) (including one CDC and one unclassified)]. Regarding nccRCC patients, 13% had PD-L1 ≥ 1%. For ccRCC patients, prior systemic therapy was excluded, but nccRCC patients with prior therapy with one TKI (not MET-targeting TKIs nor ICIs) were included.

There were two ccRCC cohorts: cabozantinib 40 mg and cabozantinib 60 mg. The ORR was 53% for atezolizumab + cabozantinib 40 mg and 58% for atezolizumab + cabozantinib 60 mg, with 3% and 11% CR, respectively. For nccRCC, there was only one cohort with atezolizumab + cabozantinib 40 mg and the ORR was 31%, all with confirmed PR. Responses were independent of the subtype or PD-L1 status. PRCC had the highest ORR (47%), followed by other histologic subtypes (25%) and chRCC (11%) In the nccRCC cohort, median PFS was 9.5 months and OS results are pending (December 2022) (39) (**Table 2**).

The main group within nccRCC was PRCC, which presented a similar ORR with cabozantinib 40 mg + atezolizumab as ccRCC (47% vs. 53%, respectively). When chromophobe histology is included in the analysis, the ORR in the nccRCC cohort is reduced to 31%. Overall, the nccRCC population had a better ORR with the combination than with TKIs alone (including cabozantinib), knowing that the ORR obtained in retrospective and early-phase studies with TKIs was approximately 27%–37% (8, 50–52).

The results of nccRCC on this study are similar to those obtained in the nccRCC cohort of the atezolizumab + bevacizumab trial (ORR 26%), but, in contrast to the atezolizumab + bevacizumab trial, the responses obtained in COSMIC-021 were independent of the PD-L1 status. Thus, PD-L1 did not seem to be a good predictor of ICI/TKI response in patients with nccRCC, although more data are required as the population and PD-L1 status were heterogeneous in the different studies.

##### Nivolumab + cabozantinib

One of the ICI + TKI combinations previously evaluated in ccRCC patients was nivolumab + cabozantinib; the CheckMate 9ER trial evaluated it in ccRCC patients, showing benefits in the ORR (p<0.001), PFS (HR 0.51 p<0.001), and OS (HR 0.60 p=0.001) with the combination compared with sunitinib alone (21).

Following these findings, a prospective phase II study was carried out to assess the ORR in patients with nccRCC receiving nivolumab + cabozantinib treatment (NCT03635892). The study had two cohorts with 47 patients: Cohort 1 (40 patients) included 32 PRCC (80%), 6 unclassified (15%), and 2 translocation-associated subtypes (5%), and Cohort 2 included 7 chRCC subtypes. Prior treatment was received by 35% of Cohort 1 and 29% of Cohort 2. The percentage of patients with positive PD-L1 was not reported. The initial results were published in 2022.

Median follow-up was 13.1 months. The ORR of Cohort 1 was 47.5% (47% in PRCC, 50% in unclassified (three of six patients), and 50% in the translocation-associated subtype (one of two patients)]. Median PFS was 12.5 months, and median OS was 28 months. In Cohort 2, no responses were observed and median PFS was not calculated due to the low number of patients (40) (**Table 2**).

The results of this study could be compared with those obtained in the COSMIC-021 trial. In both studies, PRCC was the most frequent histology, with an ORR of 47% and a median PFS of ±10 months (9.5 months with atezolizumab + cabozantinib and 12.5 months with nivolumab + cabozantinib) (39, 40).

Further studies in a larger number of patients would be necessary to detect any relevant differences when combining cabozantinib with one ICI or another.

##### Durvalumab + savolitinib

The dysregulation of MET appears to play a crucial role in PRCC pathogenesis and decreases the efficacy of TKI therapies. The SAVOIR phase III randomized trial evaluated savolitinib (MET-specific TKI) vs. sunitinib in PRCC. Although savolitinib did not significantly increase PFS (HR 0.71 p = 0.31) or OS (HR 0.51 p=0.11), the MET-specific TKI presented a better ORR than sunitinib (27% vs. 7%) (53). Other investigations and preliminary studies suggested that simultaneous MET and PD-L1 inhibition might have potential benefits (54).

The CALYPSO trial (NCT02819596) was a clinical study evaluating savolitinib in combination with durvalumab (anti-PD-L1) in RCC divided into two cohorts. The first included patients with ccRCC or with sarcomatoid differentiation and had four possible treatment arms: durvalumab, savolitinib, durvalumab and savolitinib, or durvalumab and tremelimumab. The second cohort included patients with PRCC and only one treatment arm with durvalumab + savolitinib was possible. Previous treatment was permitted.

For the nccRCC cohort, median follow-up was 26.8 months, the ORR was 29%, median PFS was 4.9 months, and median OS was 12.3 months. The evaluation of *MET* alterations (chromosome 7 gain/*MET* or *HGF* amplification/MET kinase domain mutations) was included in the analysis. Of these patients, 34% had MET-driven disease and their ORR was 57%, with a median PFS of 10.5 months and a median OS of 27.4 months (41) (**Table 2**).

The savolitinib + durvalumab combination demonstrated a better ORR in patients with MET dysregulation in comparison with savolitinib alone. Additionally, the considerable increase in the ORR in patients with PRCC and MET abnormalities makes its molecular study relevant in these patients.

## Data according to histology

### Papillary

PRCC is the most clinically evaluated nccRCC subtype. These are tumors that can originate from distal or proximal convoluted tubules, and their behavior is less aggressive than clear cell tumors.

Two decades ago, distinguishing papillary type 1 and type 2 RCC was proposed due to a morphology and the molecular differences of these variants. This classification in type 1 and 2 was also included in the WHO classification of the renal tumors since 2004 (55), but recent molecular studies suggest that type 1 and 2 papillary subgroups may not constitute a single well-defined kind (54). For this reason, in the latest WHO 2022 classification, this division is no longer recommended (3).

It was difficult to compare the results of the different studies in this histological subtype due to the heterogeneity of the sample. The PAPMET trial compared four possible treatments (crizotinib, savolitinib, sunitinib, and cabozantinib) in PRCC, showing improvements in ORR, PFS, and OS with cabozantinib vs. sunitinib: ORR 24% vs. 3%, median PFS 9 vs. 5.6 months and median OS 20 vs. 16.4 months (14).

Prospective studies with ICIs in monotherapy (Keynote-427 with pembrolizumab and Checkmate-374 with nivolumab) show differences in their PRCC population. The Keynote-427 trial included a higher percentage of patients with papillary tumors (71.5%, n = 118), none of whom had been previously treated, and CheckMate-374 included fewer patients with PRCC (54.5%, n = 24), of whom 34.1% had been previously treated. Due to this, the ORRs are very heterogeneous (28.8% with pembrolizumab and 8.33% with nivolumab) (31, 33). All considered, it was difficult to compare these results with ICI monotherapy.

Regarding combination therapies, the ORR was similar in the atezolizumab + cabozantinib (47%) and nivolumab + cabozantinib (47%) trials, being higher than that obtained with durvalumab + savolitinib (29%). The comparison was very different when only the MET alteration group was evaluated in the durvalumab + savolitinib trial, obtaining an ORR of 57%. The combination with fewer responses was atezolizumab + bevacizumab (25%) (39–41). Cabozantinib has multikinase action, blocking the MET receptor as well as other receptor tyrosine kinases. The fact that cabozantinib presented a better response than savolitinib, a MET-specific TKI, in the overall PRCC population where the status of the *MET* gene is often unknown suggested that other molecular alterations were involved in the development of papillary tumors, independent of alterations in the *MET* gene.

### Chromophobe

chRCC is the third most frequent subtype within nccRCC and originates in the distal nephron (56). They are usually not very aggressive tumors, and, even at an advanced stage, survival is greater than in other subtypes (57). chRCC is known as a “cold tumor,” referring to its low immunogenicity (58). This histological type presents a low percentage of PD-L1 expression, and some series found a positive PD-L1 in only 5% of patients with chromophobe tumors (16).

In this nccRCC subtype, there was less evidence of benefit with immunotherapy. The responses obtained were normally worse than in other subtypes, and studies with ICIs in monotherapy and combination ICI + ICI showed poorer response rates (30, 31, 33, 37). Regarding ICI+ TKI combinations, the results continued to be mediocre. ORRs were 0% for nivolumab + cabozantinib: 11% (one of nine patients) for atezolizumab + cabozantinib and 10% (1 out of 10 patients) for atezolizumab + bevacizumab (38–40).

Therefore, in these patients, the highest response rate continues to be that observed with TKIs or mTOR. The ASPEN study, comparing everolimus vs. sunitinib, presented a higher ORR (33% vs. 10%) and median PFS (11.4 vs. 5.5 months) with everolimus (11), and the ESPN and RECORD-3 studies confirmed the benefit of mTOR in chRCC patients (10, 12).

Although these patients are scarcely represented in studies, immunotherapy does not seem to be the best option and it would be necessary to carry out more targeted studies, which is difficult because of the low incidence.

### Collecting ducts

CDC exhibits a more aggressive behavior than other histologies due to its pathological resemblance to urothelial tumors. Therefore, treatment with chemotherapy (platinum + gemcitabine) falls within the recommendations for the management of CDC patients, with an ORR, median PFS, and median OS approximately 26% and 7.1 and 10.5 months, respectively (59). These results improved when chemotherapy was combined with bevacizumab in the BEVABEL trial (median PFS 15.1 months and median OS 27.8 months) (60). With the appearance of TKIs, they were assessed as to whether they improved the evolution of these patients. The prospective phase II trial with sunitinib, where 11% of patients had CDC (six patients), reported an ORR of 0% and a PFS of 3.1 months in this subgroup (61). Another prospective phase II study with cabozantinib included 23 patients with CDC histology who presented an ORR of 35% and a median PFS of 6 months (62).

According to current knowledge of tumor biology, CDC patients are characterized by the inactivation of genes involved in oxidoreductase activity, pyruvate metabolism, and the tricarboxylic acid cycle. These changes translate into increased lymphocyte infiltration into the tumor (63). In addition, CDC patients have a higher percentage of PD-L1 expression compared with PRCC and chRCC, which could be present in up to 20% of collecting duct tumors (16).

These findings motivated the assessment of immunotherapy in this subtype. In the case of monotherapy studies, retrospective and prospective nivolumab trials enrolled one or four patients with CDC in their population, with an ORR of approximately 25% (30, 32). In the combination studies, nivolumab + cabozantinib did not include CDC patients, and the atezolizumab + cabozantinib study only included one patient (39, 40). In the case of the atezolizumab + bevacizumab trial, five CDC patients were included, with an ORR of 40% (38), and, for ipilimumab + nivolumab, the CheckMate-920 trial only included two patients (37).

Knowing that this histology could potentially benefit from immunotherapy treatment, it is important to carry out more prospective studies that include a larger sample of patients with CDC and, thus, be able to confirm this hypothesis.

### Unclassified

The unclassified subtype is present in 2%–6% of nccRCC (64). This subtype includes those tumors that do not present any histological characteristics of the other subtypes and have some degree of undifferentiated tumor. Therefore, the diagnosis of this subtype is *via* exclusion; however, some molecular characteristics of the unclassified subtype are known, for example, the loss or mutation of the *NF2*, *SETD2*, *BAP1*, *KMT2C*, or *MTOR* genes (65).

These tumors present an aggressive behavior, their management being complicated since there are no studies in this subtype. Treatments with TKIs and mTOR have shown a low response rate, with ORRs between 0% and 13%, and median PFS between 4.7 and 11.5 months (10, 11, 50).

With the arrival of immunotherapy, patients with unclassified subtypes have shown encouraging results, with the ORR increasing to 25%–36% in the nivolumab trials or 34.6% in the pembrolizumab trial (30, 32, 33).

Prospective studies with combination ICI + TKI therapy also showed promising response rates. Direct data for this subtype were provided by the nivolumab + cabozantinib trial, which included six patients with an ORR of 50%, and the atezolizumab + bevacizumab study, which included nine patients with an ORR of 33% (38, 40). The atezolizumab + cabozantinib trial only included one patient with this subtype (39). On the contrary, the combination of ICI + ICI presented a worse response rate than the combination of ICI + TKI, with an ORR of 18.18% in Checkmate-920, which included 22 patients (37). It would be interesting to continue studying this tumor subtype and identify biomarkers that help us understand the mechanism of action of immunotherapy in this tumor and recognize which patients would benefit more from ICI + TKI or ICI + ICI.

## Sarcomatoid linage

According to the 2022 WHO anatomopathological classification, sarcomatoid differentiation is not defined as another subtype but rather as a type of histological differentiation that may be present in other types of kidney tumors, both in ccRCC and nccRCC. These cells are differentiated at the histological and molecular level, with more frequent mutations in these patients, such as mutations in *p53*, Von Hippel–Lindau, *CDKN2A*, *NF2*, *PBRM1*, *SETD2*, *PTEN*, *ARID1A*, or *BAP1* (66, 67). Sarcomatoid differentiation is present in 15%–20% of tumors and, as it confers a worse prognosis, it is important to know if a tumor contains this type of differentiation (68, 69).

Establishing a standard treatment for patients with sarcomatoid differentiation has been the subject of discussion for a long time, and no consensus has been reached so far. Initially, these patients were included in the TKI trials for ccRCC tumors, showing a more torpid evolution, with an ORR of 21%, a median PFS of approximately 4.5 months, and a median OS of approximately 10.4 months (70), maybe because patients with sarcomatoid differentiation presented greater resistance to angiogenic therapy (71, 72). Later, with the development of ICIs, these patients showed better results with these therapies, perhaps because of the high PD-L1 expression described in sarcomatoid tumors.

Recent trials with combination therapies for ccRCC continued to evaluate the ORR in sarcomatoid differentiation. The phase III CheckMate-214 trial compared ipilimumab + nivolumab vs. sunitinib in patients with ccRCC. In the combination arm, 12.22% of patients had sarcomatoid differentiation. This subgroup had a higher percentage of positive PD-L1 in comparison with patients without sarcomatoid differentiation (47% vs. 26%). Patients with intermediate/poor-risk disease and sarcomatoid differentiation presented a better ORR with the combination vs. sunitinib (60.8% vs. 21.3% p ≤ 0.0001) with a higher CR rate (18.9% vs. 3.1% respectively). Additionally, patients with sarcomatoid differentiation presented better median PFS (26.5 vs. 5.1 months HR 0.54 p = 0.0093) and median OS (not reached vs. 14.2 months HR 0.45 p = 0.0004) with immunotherapy than with TKIs. In this trial, ORR with ipilimumab + nivolumab was higher for patients with sarcomatoid differentiation than patients without; despite this, PFS was lower, given the aggressiveness of the tumors with sarcomatoid differentiation (73, 74).

The response with the ICI + TKI combination in ccRCC patients with sarcomatoid differentiation has been evaluated in different ccRCC trials (**Table 3**). Five relevant trials in ccRCC evaluated the response to combined treatment in patients with sarcomatoid differentiation (Immotion-151, Javelin-101, Keynote-426, Checkmate-9ER, and CLEAR). These trials detected that responses in ccRCC patients with sarcomatoid differentiation ranged from 36.6% to 61% (75–80).

**Table 3:** Sarcomatoid differentiation.

Treatment	Histology	Type of trial	sarcomatoid population (N)	ORR	PFS(months)	OS (months)
Nivolumab Checkmate-374 (31)	nccRCC	Prospective Single arm	N= 4/44 (9.1% ofpopulation)	50%	Not reported	Not reported
Pembrolizumab Keynote-427 Cohort B (33)	nccRCC	Prospective Single arm	N= 38/165 (23% of population)	42%	6.9	25.5
Ipilimumab+Nivolumab Checkmate-920 (37)	nccRCC	ProspectiveSingle arm	N= 15/ (28.8% ofpopulation)	35.7%	Not reported	Not reported
Ipilimumab+Nivolumab Checkmate 214 (75, 76)	ccRCC	Prospective Randomized	Overall N= 145/1096 (13.2% of population) Combo:N= 74/425 (17.4%) Sunitinib:N=65/422 (15.4%)	Combo: 60.8%p=< 0.0001 Sunitinib:21.3%	Combo: 26.5HR 0.54 (95%CI, 0.3–0.9) Sunitinib:5.1	Combo: Not reachedHR 0.45 (95%CI, 0.3–0.7) Sunitinib:14.2
Atezolizumab+Bevacizumab IMmotion 151 (77, 78)	ccRCC	Prospective Randomized	Overall: N= 142/915 (15.5% ofpopulation) Combo: N= 68/461 (14.7%) Sunitinib:N= 74/461(16%)	Combo: 49% Sunitinib: 14%	Combo: 8.3HR 0.52 (95%CI 0.34–0.79) Sunitinib: 5.3	Combo: 21.7HR 0.64 (95%CI 0.41- 1.01) Sunitinib: 15.4
Avelumab+Axitinib Javelin 101 (79)	ccRCC	Prospective Randomized	Overall N= 108/886 (12.2%) Combo: N= 47/442 (10.6%) Sunitinib: N= 61/444(13.7%)	Combo: 46.8% Sunitinib: 21.3%	Combo: 7.0 HR 0.57 (95% CI,0.325-1.003) Sunitinib:4.0	Not reported any arm HR 0.57 (95% CI,0.325-1.003)
Pembrolizumab+Axitinib Keynote 426 (80)	ccRCC	Prospective Randomized	Overall N= 105/861 (12.2% of population) Combo: N= 51/432 (12%) Sunitinib: N= 54/429(13%)	Combo: 58.8% Sunitinib:31.5%	Combo: NR HR 0.54 (95% CI0.29-1.00) Sunitinib:8.4	NR any arm HR 0.58 (95% CI 0.21-1.59)
Nivolumab+Cabozantinib Checkmate 9ER (81)	ccRCC	Prospective Randomized	Overall N= 75/651 (11.5% of population) Combo: N= 34/323 (10.9%) TKI: N= 41/328 (12.9%)	Combo: 55.9% Sunitinib: 22%	Combo: 10.9 HR 0.39 (95% CI0.22-0.70) Sunitinib:4.2	Combo: NRHR 0.36 (95% CI 0.16-0.82) Sunitinib:19.7
Pembrolizumab+Lenvatinib CLEAR (82)	ccRCC	Prospective Randomized	Overall N= 49/712 (6.8% ofpopulation) Combo: N= 28/355 (7.9%)Sunitinib: N= 21/357 (5.9%)	Combo 60.7% Sunitinib:23.8%	Combo 11HR 0.39 (95% CI0.18–0.84) Sunitinib:5.5	NR any arm HR 0.91 (95% CI 0.32–2.58)
Atezolizumab+Bevacizumab (38)	ccRCC and nccRCC	Prospective Single arm	Overall N= 26/60 (43% of population) ccRCCN= 18/18 (100%) nccRCCN= 8/42 (21%)	ccRCC with sarcomatoid differentiation: 50% nccRCC 38%	Not reported	Not reported
Atezolizumab+Cabozantinib COSMIC-021 (39)	ccRCC and nccRCC	Prospective Single arm	Overall N= 15/102 (14.7% of population) ccRCCN= 11/70 (15.7%) nccRCC N= 4/32 (13%)	Not reported	Not reported	Not reported

NA, not applicable; NR, not reached; OS, overall survival; PFS, progression-free survival; ORR, overall response rate; nccRCC, non clear cell renal carcinoma; ccRCC, clear cell renal carcinoma. HR, Hazard ratio; CI, Confidence interval

Tumors with a sarcomatoid component have been evaluated in different TKI trials over the years and presented an ORR of around 21% (71). This result was enhanced with immunotherapy and could be justified by the high expression of PD-L1 described in sarcomatoid tumors. Monotherapy ICI trials for nccRCC with sarcomatoid differentiation showed an ORR of around 42-50%. ICI+ICI and ICI + TKI combinations improved that result. When referring to the ICI combinations for nccRCC, the results with ipilimumab + nivolumab are higher than with the ICI monotherapy, with ORR rates of around 35-43% (35, 37). Regardong ICI + TKI trials for nccRCC, the sarcomatoid population was too limited to draw clear conclusions. The atezolizumab + bevacizumab trial for nccRCC included eight patients with sarcomatoid differentiation. These patients presented a higher ORR than patients without sarcomatoid differentiation (38% vs. 26%). The atezolizumab + cabozantinib trial included four patients with sarcomatoid differentiation and only one patient presented a response, and the last trial was the nivolumab + cabozantinib study, which included only two patients with sarcomatoid differentiation and their responses were not reported (38–40).

In the case of nccRCC, the sarcomatoid population was also evaluated in the different trials, despite their low representation. On the one hand, studies with ICIs showed higher response rates in patients with sarcomatoid differentiation than those without (**Table 3**). Checkmate-374, with nivolumab, showed an ORR of 50% in nccRCC with sarcomatoid differentiation (31). The pembrolizumab trial had two cohorts (nccRCC and ccRCC) and showed an ORR of 42% for nccRCC with sarcomatoid differentiation and 63.6% for ccRCC with sarcomatoid differentiation (33, 77, 81). These results were better than those obtained for the total population of these studies. When referring to the ICI combinations for nccRCC, the results with ipilimumab + nivolumab should be highlighted (**Table 3**), with ORR rates of approximately 35%–43% (35, 37).

In addition, there were ICI + TKI trials for nccRCC, where the sarcomatoid population was too limited to draw clear conclusions. The atezolizumab + bevacizumab trial for nccRCC included eight patients with sarcomatoid differentiation. These patients presented a higher ORR than patients without sarcomatoid differentiation (38% vs. 26%). The atezolizumab + cabozantinib trial included four patients with sarcomatoid differentiation, and only one patient presented a response. The last trial was the nivolumab + cabozantinib study, which included only two patients with sarcomatoid differentiation, and their responses were not reported (38–40).

In general, the combination therapy studies share a benefit in the ORR for patients with sarcomatoid differentiation compared with patients without this histology. Even so, this subgroup of patients continues to have worse OS than patients without this differentiation. Therefore, in patients with sarcomatoid differentiation, the recommendation is to perform a PD-L1 study and, whenever possible, prioritize treatment with immunotherapy given the higher rate of response observed in these patients.

## Discussion

Approximately 20% of renal cancers are nccRCC subtypes; fewer studies, initially aiming to improve treatment, have been carried out in this population. For this reason, treatments for nccRCC are based on those established for ccRCC, even though the nccRCC subtypes were not included in the pivotal ccRCC clinical trials. Thanks to recent advances in understanding the biology of neoplastic cells, it is now known that these cells can escape immunological responses. Therefore, treatments with ICIs (anti-PD-1, anti-CTLA-4, or anti-PD-L1) could improve the response in these patients and immunotherapy treatment is currently being evaluated, both alone and in combination with targeted therapies, specifically with VEGF pathway inhibitors and mTOR inhibitors.

As for the prospective trials with an ICI + ICI combination, the direct comparison between ipilimumab + nivolumab trials for nccRCC (Checkmate-920) and ccRCC (Checkmate-214) is limited due to the different clinical characteristics, independent of histological types, since the Checkmate-920 study targeted patients with characteristics that conferred a worse prognosis (low Karnofsky grading and brain disease) (24, 37). Despite this, both the 1 ccRCC cohort of the Checkmate-920 study and the Checkmate-214 study for ccRCC show better results than the nccRCC patients in the Checkmate-920 study.

Regarding the ICI + target therapy combination, the population included in the different studies is small (42 nccRCC patients in the atezolizumab + bevacizumab trial, 32 for atezolizumab + cabozantinib, 47 for nivolumab + cabozantinib, and 41 for durvalumab + savolitinib). In all of these trials, the most represented histology was PRCC, but the proportion of PRCC compared with other subtypes in the studies was different and made it difficult to draw any clear conclusions. For PRCC, combinations containing a MET-specific TKI (savolitinib) or a multi-TKI targeting MET (cabozantinib) had the best response rates, achieving an ORR of 47% with atezolizumab + cabozantinib, 47% with nivolumab + cabozantinib, and 29% or 57% with durvalumab + savolitinib depending on the *MET* gene status (the response is greater in patients with alterations in the *MET* gene) (38–41).

The fact that patients without known *MET* alterations had a better response with non-specific TKIs suggested that there are other pathways involved in the carcinogenesis of these tumors. This should be taken into account when considering the TKI for PRCC patients in case the *MET* gene shows no alteration or is unknown.

Regarding the other histologies, their representation was heterogeneous in the studies. In the combination studies, the nivolumab + cabozantinib trial did not include CDC patients and the atezolizumab + cabozantinib trial included only one patient with this histology (39, 40). In the case of atezolizumab + bevacizumab, CDC was represented by five patients with an ORR of 40% (38), and the Checkmate-920 trial with ipilimumab + nivolumab only included two CDC patients (37). It is known that CDC is related to high lymphocyte infiltration into the tumor and higher PD-L1 expression compared with other subtypes. This should motivate a more direct study of the benefit of immunotherapy in these patients.

In the case of unclassified subtypes, ICI monotherapy studies showed an ORR of approximately 25%–36%. Regarding combination therapies, the trials providing direct data for unclassified subtypes were the nivolumab + cabozantinib (which included six patients with an ORR of 50%) and atezolizumab + bevacizumab studies (which included nine patients with an ORR of 33%) (38, 40). Considering that the responses obtained with other treatments, such as TKIs or mTOR, range from 0% to 13%, immunotherapy does seem to have a clear benefit in this patient subtype.

chRCC is represented in all combination therapy trials with a low ORR, ranging from 0% with nivolumab + cabozantinib to 11% with atezolizumab + cabozantinib. The fact that this subtype is associated with low PD-L1 expression could explain the poorer response to ICI therapies. Thus, immunotherapy does not seem the best option and it would be necessary to carry out more targeted studies, despite the low incidence.

In the case of tumors with a sarcomatoid component, they have been evaluated in different trials and presented an ORR with TKIs of approximately 21% (70). This result was enhanced with immunotherapy and could be justified by the high expression of PD-L1 described in sarcomatoid tumors. On the one hand, monotherapy trials showed an ORR of approximately 42%–50%. On the other hand, evaluating the results of ICI + ICI and ICI + TKI, the ICI + TKI combinations were also more effective in ccRCC with sarcomatoid differentiation than in nccRCC with sarcomatoid differentiation [with limited data on nccRCC with sarcomatoid differentiation due to its limited inclusion in the studies (from one to eight patients in the different trials)] and the ICI + TKI combination for ccRCC with the highest ORR in ccRCC was pembrolizumab + lenvatinib (ORR 60.7%) (80). Again, the ORR with ICI + ICI, ipilimumab + nivolumab, was higher for ccRCC with sarcomatoid differentiation (ORR 60.8%) than for nccRCC with sarcomatoid differentiation (ORR 43%) (73, 74).

The results of the role of PD-L1 were heterogeneous. In the case of ipilimumab + nivolumab or atezolizumab + bevacizumab, the expression of PD-L1 was related to a higher ORR. In contrast, the study with atezolizumab + cabozantinib showed no differences in response based on PD-L1 expression. In the case of nivolumab + cabozantinib, this biomarker was not evaluated. If PD-L1 expression is evaluated according to histology, the subtypes with higher expression present better responses to immunotherapy (unclassified, CDC, and sarcomatoid differentiation) than subtypes with lower PD-L1 expression. According to all these data, it can be concluded that more studies will be necessary to clarify the role of PD-L1 in nccRCC.

Finally, it should be noted that the response to immunotherapy within the nccRCC group was heterogeneous. Those histological subtypes that seemed to present greater benefit were CDC, PRCC, and unclassified, with chRCC presenting the lowest response. The need to a) conduct trials in a larger number of patients with different histologies, b) continue studying the role of PD-L1, and c) search for new biomarkers that help predict response to treatment continues to make this topic a necessary question for constant review (82) and make it an important point of future research, in order to continue improving the treatment of these patients.

## Author contributions

CC, SS, and EG have carried out an exhaustive study of the published bibliography on the subject in question, in addition to writing and discussing the conclusions obtained. The rest of the authors have contributed to the literature search and final correction of the current review. All authors contributed to the article and approved the submitted version.
